# Intestinal Polyamine Metabolism and Mucosal Barrier in Ningxiang and DLY Piglets: Differential Responses to ETEC Challenge

**DOI:** 10.3390/ani16091336

**Published:** 2026-04-27

**Authors:** Yunfang Song, Luya Feng, Yunlong Meng, Hao Cheng, Jing Wang, Yao Yue

**Affiliations:** 1 Department of Endocrinology, The Affiliated Hospital of Inner Mongolia Medical University, Huhhot 010030, China; 18084080964@163.com (Y.S.); m13297460179@163.com (L.F.); 15838295579@163.com (Y.M.); 2 Hunan Provincial Key Laboratory for the Products Quality Regulation of Livestock and Poultry, College of Animal Science and Technology, Hunan Agricultural University, Changsha 410128, China; jingwang023@hunan.edu.cn; 3 Yuelushan Laboratory, Changsha 410128, China; 4 Institute of Synthetic Biology Industry, Hunan University of Arts and Science, Changde 415000, China; chenghao19970316@163.com; 5 National Center of Technology Innovation for Dairy, Huhhot 010111, China

**Keywords:** Ningxiang piglets, intestinal mucosal barrier, polyamines, immune function, *Escherichia coli*

## Abstract

This study compared intestinal polyamine metabolism and barrier function between Ningxiang (NX) and Duroc × Landrace × Yorkshire (DLY) piglets under baseline conditions and following ETEC challenge. Experiment 1 (baseline, *n* = 12/breed) assessed colonic barrier integrity, immune status, polyamines, and microbiota. Experiment 2 (ETEC challenge, *n* = 8/group/breed) evaluated responses to oral ETEC (10^9^ CFU) over 3 days. Under baseline conditions, NX piglets showed superior barrier integrity, higher goblet cell numbers and mucin 2 (MUC2) protein expression, and lower plasma levels of intestinal permeability markers—diamine oxidase (DAO), D-lactate (DLA), and endotoxin (ET)—compared with DLY piglets. NX piglets also exhibited reduced colonic pro-inflammatory cytokine levels (*IL-6* and *IL-1β*) and higher expression of immune-related markers (CD3, CD68, and IgA) versus DLY piglets. In contrast, DLY piglets displayed more active microbial polyamine metabolism in the colon, with higher concentrations of putrescine, spermidine, and spermine, as well as increased ornithine decarboxylase (ODC) expression. 16S rRNA sequencing revealed greater microbial diversity and enrichment of taxa (*Muribaculaceae_unclassified*, *Prevotella*) in NX piglets, whereas DLY piglets showed enrichment of polyamine-associated genera (*Collinsella*, *Veillonella*). Following the ETEC challenge, DLY piglets displayed pronounced polyamine upregulation, including elevated polyamine levels and ODC1 expression. Conversely, NX piglets maintained more stable polyamine metabolism, higher expression of tight junction proteins (ZO-1 and occludin), lower plasma permeability markers, reduced pro-inflammatory cytokine expression (*IL-6*, *IL-1β*, *IL-22*), and increased anti-inflammatory *IL-10* expression. Collectively, these findings demonstrate that NX piglets possess superior intestinal barrier integrity and immune maturity, while DLY piglets exhibit a more active but stress-responsive polyamine metabolic phenotype. The divergent metabolic and immune responses to ETEC challenge underscore the distinct strategies employed by these two breeds in maintaining gut homeostasis. These findings provide preliminary insights that may inform future breeding strategies aimed at enhancing intestinal health and disease resistance in pigs, pending validation in broader genetic backgrounds and mechanistic studies.

## Simple Summary

The intestinal mucosal barrier is the first line of defense against enteric pathogens, yet its functional differences among pig breeds remain poorly understood. This study explores how Ningxiang (NX) and Duroc × Landrace × Yorkshire (DLY) piglets differ in intestinal immune status and microbial polyamine metabolism, both under normal conditions and following infection with enterotoxigenic *Escherichia coli* (ETEC). Our findings show that NX piglets have a stronger colonic mucosal barrier and a more mature local immune environment, characterized by increased goblet cells, higher mucin expression, and reduced systemic inflammation. In contrast, DLY piglets exhibit a more active microbial polyamine metabolism, which becomes even more pronounced after ETEC infection—potentially reflecting a compensatory response to intestinal injury. By linking breed-specific microbial metabolic traits with host immune resilience, this study highlights the complex interplay between host genetics, gut microbiota metabolism, and susceptibility to infection. These findings provide a scientific basis for developing breed-targeted nutritional or therapeutic strategies to improve gut health and disease resistance in swine production.

## 1. Introduction

Due to immature intestinal development and a weakened immune system, weaning piglets are particularly sensitive to changes in feed and husbandry environment, making them susceptible to intestinal dysfunction and diarrhea [1]. This not only hinders piglet growth efficiency but also reduces survival rates, resulting in substantial economic losses to the livestock industry. Therefore, understanding the mechanisms that support intestinal health in weaned piglets is of great practical importance.

The intestinal mucosal barrier comprises four main components: (1) the physical barrier formed by tight junction proteins (e.g., ZO-1, occludin), which regulate paracellular permeability; (2) the chemical barrier consisting of the mucus layer (mainly MUC2 secreted by goblet cells); (3) the immune barrier involving immune cells (CD3^+^ T cells, CD68^+^ macrophages) and secretory IgA; and (4) the microbial barrier composed of commensal bacteria that prevent pathogen colonization and maintain mucosal homeostasis [2,3]. Disruption of these barriers increases plasma levels of permeability markers such as diamine oxidase (DAO), D-lactate (DLA), and endotoxin (ET), and elevates pro-inflammatory cytokines (e.g., *IL-6*, *IL-1β*) [4].

Notably, the integrity of this intestinal barrier varies significantly across pig breeds. Compared to Duroc × Landrace × Yorkshire (DLY) pigs, Chinese native breeds are characterized by superior traits, including tolerance to coarse feed, low diarrhea rates, and strong disease resistance [5,6]. Ningxiang pigs (NX), a local breed from Hunan Province, China, originate from hilly regions and are adapted to diverse climatic and feeding environments, enabling robust growth under both high and low temperatures, as well as humid and dry conditions [7]. The long-term consumption of high-fiber diets by NX pigs has contributed to the development of their highly mature intestinal immunity [8]. A previous study found a more diverse bacterial community in the colon of NX pigs compared to the large white pigs [9], and this rich gut microbiota effectively resists the invasion by harmful bacteria, enhancing intestinal immunity [10]. Thus, significant differences likely exist in intestinal microbial structure and metabolism between NX and DLY pigs.

Among the various metabolites that regulate intestinal barrier function, polyamines (putrescine, spermidine, and spermine) have emerged as key players [11,12,13]. Polyamines are synthesized from arginine via ornithine decarboxylase (ODC), the rate-limiting enzyme in the polyamine biosynthesis pathway [14,15,16]. Polyamines are noted to be crucial for regulating cell growth and proliferation, maintaining DNA negative charge stability, and facilitating RNA transcription and protein synthesis, as well as modulating cell apoptosis and immune responses [17]. Notably, dietary supplementation with spermine has been shown to improve the villus height and enhance the expression of adhesion junction in weaned piglets, thereby enhancing the intestinal barrier function and ensuring the normal maintenance of intestinal permeability [18,19]. Furthermore, arginine levels, the precursor for polyamine synthesis, have been reported to be significantly different between 165-day-old NX and DLY pigs in serum metabolomic analyses [20]. A comparative study of obese versus lean pig breeds also revealed enhanced arginine metabolism and enrichment of the γ-glutamyl-L-putrescine pathway in obese pigs [21]. These findings suggest that breed-dependent differences in polyamine metabolism may contribute to distinct intestinal barrier phenotypes.

In addition to polyamine metabolism, our recent studies have shown that NX pigs have unique microbial properties associated with their phenotype [22,23,24]. Based on these lines of evidence, we hypothesized that NX piglets possess a more robust colonic mucosal barrier than DLY piglets, and that this difference is associated with breed-specific patterns of colonic polyamine metabolism. To test this hypothesis, the present study compared intestinal barrier function, polyamine levels, and polyamine-related enzyme expression between NX and DLY piglets under both baseline (healthy) conditions and following enterotoxigenic *Escherichia coli* (ETEC) challenge. Specifically, we aimed to: (1) characterize breed differences in colonic mucosal barrier integrity and immune status; (2) compare colonic polyamine metabolism between the two breeds; and (3) evaluate their differential responses to ETEC infection. The findings provide insights into the mechanisms underlying the disease resistance of NX piglets and may inform breeding strategies aimed at improving intestinal health in pigs.

## 2. Materials and Methods

### 2.1. Animals and Experimental Design

The animal study protocol was reviewed and approved by the Institutional Animal Care and Use Committee of Hunan Agricultural University (approval number: CACAHU 2021-01106, Changsha, China).

Trial 1. A total of 24 male piglets were randomly divided into two groups: Duroc × Landrace × Yorkshire (DLY) weaned piglets (8.32 ± 0.17 kg) at 28 days of age, and Ningxiang (NX) weaned piglets (8.54 ± 0.05 kg) at 28 days of age were obtained from two local commercial swineherds, respectively. Each group had twelve replicate pens, and one piglet per pen was housed individually. All piglets had free access to water and feed, with humidity stabilized at 60~65% and temperature maintained around 20~27 °C. The formula of the basic diet followed the NRC (2012) for swine nutrition [25]. The basic formula and nutritional levels are presented in Table 1. Both breeds were fed the same commercial basal diet for 7 days, and 24 piglets were slaughtered on the 8th day of the experiment period after fasting for 12 h.

Trial 2. A total of 16 male piglets were randomly divided into two groups: Duroc × Landrace × Yorkshire (DLY) weaned piglets (7.61 ± 0.20 kg) at 28 days of age, and Ningxiang (NX) weaned piglets (7.73 ± 0.34 kg) at 28 days of age were obtained from two local commercial swineherds, respectively. All 16 piglets were challenged with Enterotoxigenic *Escherichia coli* (ETEC). All piglets were given free access to water and were fed the same commercial basal diet for 10 days. Subsequently, each piglet received ETEC bacterial suspension (10 mL, 4 × 10^9^ CFU/mL) via gavage once per day for three days. Daily clinical observations were conducted, and diarrhea rates were recorded. On the 14th day of the experiment period, piglets were slaughtered after fasting for 12 h.

### 2.2. Sampling Collection and Analysis

Blood samples (5 mL) were collected by venipuncture of the jugular vein following a 12 h food deprivation period. Blood samples were collected into heparinized vacuum tubes. Blood samples were centrifuged at 3000× *g* for 10 min at room temperature (Eppendorf 5810R, Eppendorf AG, Hamburg, Germany) to collect plasma, and then immediately stored at −80 °C for plasma enzyme-linked immunosorbent assay (ELISA) analysis. Following blood sample collection, all piglets were euthanized under anesthesia via intravenous pentobarbital sodium injection (50 mg/kg BW), followed by dissection. Contents of the proximal colon were harvested from all piglets and stored at −80 °C for subsequent 16S rDNA sequencing, untargeted metabolomic profiling, and polyamine quantification. An approximately 6 cm segment of proximal colonic tissue (starting 10 cm distal to the ileocecal junction) was immediately isolated from each piglet and rinsed with saline. This segment was then divided into two parts: one 3 cm segment was fixed in 4% paraformaldehyde for morphological examination, immunohistochemical analysis, and immunofluorescence analysis, and the other 3 cm segment was snap-frozen and stored at −80 °C for subsequent real-time quantitative polymerase chain reaction (RT-qPCR), Western blot, tissue polyamine measurement, and ELISA.

### 2.3. Histological and Immunological Staining

Colonic samples were fixed in 4% paraformaldehyde solution for 24 h at room temperature. After fixation, tissues were dehydrated through a graded ethanol series (70%, 80%, 95%, and 100% ethanol), cleared in xylene, and embedded in paraffin. Serial sections (5 μm) were cut and stained with hematoxylin and eosin (H&E) for histological examination. For goblet cell enumeration, five randomly selected, non-overlapping fields per section were captured at 400× magnification using a light microscope (Zeiss Axio Imager Z2, Carl Zeiss AG, Jena, Germany). Goblet cells were identified by their characteristic vacuolated appearance and positive staining with H&E (pale-staining cytoplasm). The number of goblet cells was counted manually using Image-Pro Plus 6.0 software (Media Cybernetics, Rockville, MD, USA) and expressed as the mean number of goblet cells per mm^2^ of colonic mucosa, as previously described [27]. All analyses were performed by an investigator blinded to the experimental groups.

Immunohistochemistry (IHC) was performed as previously described [28]. The primary antibodies used were rabbit anti-CD3 (1:100, cat. #ab16669, Abcam, Cambridge, UK), anti-CD68 (1:3000, cat. #ab303565, Abcam, Cambridge, UK) and mouse anti-IgA (1:25, cat. #ab124716, Abcam). Secondary antibodies were HRP-conjugated goat anti-rabbit IgG (1:200, cat. #31460, Thermo Fisher Scientific, Waltham, MA, USA) and HRP-conjugated goat anti-mouse IgG (1:200, cat. #31430, Thermo Fisher Scientific, Waltham, MA, USA). Following secondary antibody incubation, sections were visualized using DAB substrate and counterstained with hematoxylin. Negative controls were performed by substituting the primary antibodies with PBS. Colon tissue sections were examined under a light microscope at 400× magnification, and images were captured. Quantitative analysis of IHC staining was conducted using the mean optical density (AOD) value, which was determined with Image-J software (version 1.53, National Institutes of Health, Bethesda, MD, USA). The AOD for CD3, CD68, and IgA-positive cells was calculated according to established methodologies [29,30].

Immunofluorescence (IF) was performed as previously outlined [30]. Colon tissue sections were deparaffinized, rehydrated, and subjected to antigen retrieval by microwaving in citrate buffer (pH 6.0) for 10 min. Non-specific binding was blocked with 5% normal goat serum in PBS containing 0.1% Triton X-100 for 30 min at room temperature. Sections were separately incubated overnight at 4 °C with primary antibodies: rabbit anti-Muc2 (1:500, cat. #ab272692, Abcam, Cambridge, UK) and rabbit anti-ZO-1 (1:500, cat. #GB15195, Servicebio, Wuhan, China). After washing with PBS, sections were incubated with Alexa Fluor 488 goat anti-rabbit IgG (cat. #GB25303, Servicebio, Wuhan, China) for 1 h at room temperature in the dark. Nuclei were counterstained with DAPI for 5 min. Negative controls were performed by substituting the primary antibodies with PBS. Sections were visualized under a fluorescence microscopeat 200× magnification. Images were captured using ZEN software (version 3.0, Carl Zeiss AG, Jena, Germany). Quantitative analysis of fluorescence intensity was performed using ImageJ software (version 1.53, National Institutes of Health, Bethesda, MD, USA), and results were expressed as mean fluorescence intensity (MFI) after background subtraction.

### 2.4. Enzyme-Linked Immunosorbent Assay

Concentrations of inflammatory cytokines interleukin-6 (*IL-6*), interleukin-1 beta (*IL-1β*), and interleukin-10 (*IL-10*), as well as the ODC enzyme activity in colonic tissue, were determined using enzyme-linked immunosorbent assay kits: cat. #CSB-E06786p, cat. #CSB-E06782p, cat. #CSB-E06779p, from CUSABIO (CUSABIO Technology LLC, Houston, TX, USA); and cat. #MM-78087O1 (ODC activity) from Jiangsu Meimian Industrial Co., Ltd. (Yancheng, China). For plasma measurements, diamine oxidase (DAO, cat. #MM-0438O1, Jiangsu Meimian Industrial Co., Ltd., Yancheng, China), endotoxin (ET, cat. #MM-36368O1, Jiangsu Meimian Industrial Co., Ltd., Yancheng, China), and D-lactate (DLA, cat. #MM-3373202, Jiangsu Meimian Industrial Co., Ltd., Yancheng, China) were analyzed using a porcine ELISA kit). Detailed procedures for these assays followed the respective kit manuals closely.

### 2.5. RNA Extraction and RT-qPCR

The RNA of colon tissue was prepared using the RNeasy Kit (Qiagen, Hilden, Germany). cDNA was synthesized from total RNA according to the *Evo M-MLV* RT Kit with gDNA Clean for qPCR II (cat. #AG11711, Accurate Biotechnology (Hunan) Co., Ltd., Changsha, China). RT-qPCR was performed using the SYBR Green Premix Pro Taq HS qPCR kit (cat. #AG11701, Accurate Biotechnology (Hunan) Co., Ltd. Changsha, China) by a real-time PCR instrument (LightCycler480 II system, Roche Diagnostics, Basel, Switzerland) with LightCycler 480 Software (version 1.5.1). Beta-actin was used for reference genes to normalize the expression levels of the target gene using the 2^−ΔΔCt^ method. The primers used are listed in Table 2.

### 2.6. High-Resolution Metabolomics (UPLC-MS)

Metabolomic profiling of colonic content was conducted by Metaboprofile Co., Ltd. (Shanghai, China) using UPLC-MS/MS (Q-Exactive Plus, Thermo Fisher Scientific, Waltham, MA, USA). Sample preparation, extraction, and LC-MS parameters were performed as described in the Supplementary Methods. Raw data were processed by the service provider using their standard pipeline, including peak detection, alignment, filtering, log normalization, and metabolite identification against public databases (MoNA, Metlin) and an in-house spectral library. Orthogonal partial least-squares discriminant analysis (OPLS-DA) was performed to assess metabolic profile differences between groups. Differential metabolites were identified based on variable importance in projection (VIP) ≥ 1.0, fold change (FC) ≥ 1.0, and *p* < 0.05 (Student’s *t*-test), with false discovery rate (FDR) correction applied for multiple comparisons [31].

### 2.7. 16S rDNA Gene Sequencing

DNA was extracted from mid-colonic contents using a Stool DNA Isolation Kit (SENO Biotech Co., Ltd., Zhangjiakou, China) following the manufacturer’s protocol. All colon content samples were sequenced on the Illumina NovaSeq platform (LC-Bio Technology Co., Ltd., Hangzhou, China). Briefly, total bacterial DNA was extracted from colonic contents, followed by PCR amplification and product purification. After library preparation and quality control, sequencing was conducted. The universal forward primer 338F (5′-ACTCCTACGGGAGGCAGCA-3′) and universal reverse primer 806R (5′-GGACTACHVGGGTWTCTAAT-3′) were used to amplify the V3–V4 regions of 16S rRNA gene [32]. The paired-end data were merged using overlapping regions, followed by filtering and quality control to obtain raw data. The raw data were subsequently denoised using the Divisive Amplicon Denoising Algorithm (DADA2), and Amplicon Sequence Variants (ASVs) were employed to construct an OTU-like table. The final feature table and representative sequences were generated, facilitating further diversity analysis, taxonomic annotation, and differential analysis. Then, bioinformatics analysis was performed by QIIME (Version 1.8.0, http://qiime.org/, accessed 15 March 2022). These analyses included relative abundance at the phylum and genus levels, α-diversity and β-diversity assessments, and potential pathway analysis. Independent sample non-parametric tests were employed to compare microbial community differences between the two groups at the phylum and genus levels.

### 2.8. Intestinal Polyamines Contents Analysis

Colonic tissues (1 g) and contents (1 g) were homogenized with 0.4 mol/L perchloric acid solution (5 mL) and stored at −20 °C overnight for extraction. The samples were centrifuged at 8000× *g* for 10 min at room temperature, and the supernatant was collected. Buffered solutions (1 mL, pH = 10.6) of sodium hydroxide and saturated sodium bicarbonate were added, followed by dansyl chloride (Sigma, St. Louis, MO, USA) dissolved in acetone (10 mg/mL), all under dark conditions for a 30 min heating reaction, gently shaken and mixed every 5 min during the reaction. After the reaction was over, 5% (*v*/*v*) aqueous ammonia (1 mL) was added. The mixture was cooled, and anhydrous ether (3 mL) was employed for extraction. The ether layer was collected after 10 min of extraction and concentrated to dryness using a Vacuum Centrifugal Concentrator (SPD 130P, Thermo Fisher Scientific, Waltham, MA, USA). The residue was dissolved in methanol (1 mL), filtered through a 0.22 μm organic phase filter membrane, and prepared for analysis by High Performance Liquid Chromatography (Agilent 1200, Agilent Technologies, Santa Clara, CA, USA). The final data were normalized to tissue weight.

### 2.9. Western-Blotting

Frozen colon tissue samples were homogenized in RIPA lysis buffer (Beyotime, Shanghai, China) supplemented with a protease inhibitor cocktail (Beyotime, Shanghai, China). The lysates were collected and heated in boiling water for about 5 min. Equal amounts of protein samples were subjected to 10% sodium dodecyl sulfate–polyacrylamide gel electrophoresis and separated under appropriate conditions, then transferred onto polyvinylidene fluoride membranes. The membranes were blocked with 5% skimmed milk for 2 h at room temperature (RT), washed in TBST buffer, and incubated overnight with primary antibodies at 4 °C (ODC1, 1:800, cat. #bs-1294R, Bioss, Beijing, China; occludin, 1:1000, cat. #27260-1-AP, Proteintech, Inc., Chicago, IL, USA), followed by secondary antibodies (cat. #A21020, anti-rabbit, 1 μg/L, Proteintech, Inc., Chicago, IL, USA). After washing with TBST, the protein bands were visualized using enhanced chemiluminescence (ECL) substrate and imaged with a ChemiDoc Go Imaging System (Bio-Rad Laboratories, Inc., Hercules, CA, USA). Images were acquired using the built-in Image Lab Touch Software (version 4.0, Bio-Rad Laboratories, Inc., Hercules, CA, USA). Protein blots were quantified using Image-Pro Plus (version 6.0, Media Cybernetics, Rockville, MD, USA) and normalized to *β-actin* [33].

### 2.10. Statistical Analysis

The data were analyzed using SPSS 26.0 statistical software (for Windows, SPSS Inc., Chicago, IL, USA). Normality was verified using the Shapiro–Wilk test, and homogeneity of variances was confirmed using Levene’s test. Since all data met the assumptions of parametric tests, Student’s *t*-test was used for two-group comparisons. The mean and standard error of the mean (SEM) were used to denote error bars. Statistical significance was determined using *p*-values (* *p* < 0.05, ** *p* < 0.01, and *** *p* < 0.001). Figures were generated using Graphpad Prism 7.0 (La Jolla, CA, USA).

Multiple comparisons and pseudoreplication: For analyses involving multiple comparisons (e.g., metabolomics), the false discovery rate (FDR) was controlled using the Benjamini–Hochberg method, with adjusted q < 0.05 considered significant. To avoid pseudoreplication, the animal (*n* = number of piglets) was used as the independent experimental unit for all analyses. For histological and immunohistochemical analyses, the average value of multiple fields per animal was first calculated, and the animal was treated as the experimental unit. Spearman’s rank correlation analysis was performed by Lianchuan Bio (Hangzhou, China) to assess the relationships among colonic polyamines, intestinal barrier markers, inflammatory cytokines, and immune cell markers. Results are presented as correlation heatmaps and matrices. Statistical significance was set at *p* < 0.05.

## 3. Results

### 3.1. Differential Colonic Barrier Function Between NX and DLY Piglets

**Figure 1:**
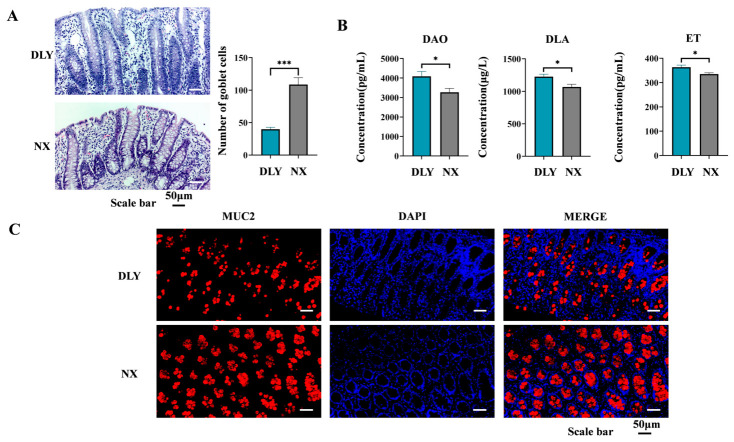
Difference in colonic mucosal mechanical barrier and permeability between NX piglets and DLY piglets. (**A**) Histological representative images of the colonic mucosa (Scale bar, 50 μm); (**B**) DAO, DLA and ET concentrations in plasma; (**C**) representative immunofluorescence images of MUC2 protein expression level in the colonic mucosa (scale bar, 50 μm). DAO: diamine oxidase; DLA: D-lactate; ET: Endotoxin; DLY: Duroc × Landrace × Yorkshire piglets; NX: Ningxiang piglets. * *p* < 0.05, and *** *p* < 0.001.

The goblet cell count in the colon of NX piglets was significantly higher (*p* < 0.01) than that in DLY piglets, approximately 2.8-fold greater (Figure 1A). The plasma concentrations of DAO, DLA, and ET were significantly lower in NX piglets than in DLY piglets (*p* < 0.05), indicating a more intact colonic mucosa in NX piglets (Figure 1B). The results of colonic immunofluorescence staining indicate that MUC2 protein expression in the colonic mucosa of NX piglets was higher than in DLY piglets (Figure 1C).

**Figure 2:**
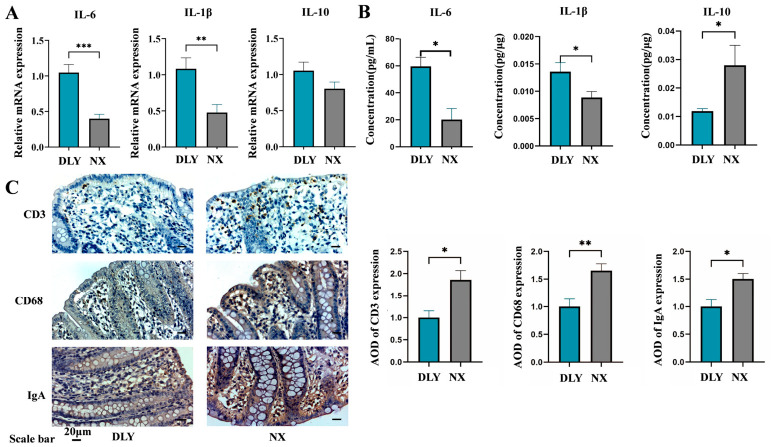
Differences in the colonic immune barrier between NX piglets and DLY piglets. (**A**) The relative mRNA expression of *IL-6*, *IL-1β*, and *IL-10* genes in the colon; (**B**) the concentrations of *IL-6*, *IL-1β*, and *IL-10* in the colon; (**C**) immunohistochemical representative images and quantitative analysis of expression levels of immune cell proteins (CD 3, CD 68, and IgA) in the colon (Scale bar, 20 μm). DLY: Duroc × Landrace × Yorkshire; NX: Ningxiang. * *p* < 0.05, ** *p* < 0.01, and *** *p* < 0.001.

The results indicated that NX piglets exhibited lower gene mRNA expression and concentration of colonic pro-inflammatory factors compared to those in DLY piglets. The relative mRNA expression levels of pro-inflammatory cytokines *IL-6* and *IL-1β* in the colon of NX piglets were significantly lower than in DLY piglets (*p* < 0.01) (Figure 2A). The concentrations of *IL-6* and *IL-1β* in the colon of NX piglets were substantially lower. In contrast, the concentration of the anti-inflammatory cytokine *IL-10* was significantly higher compared to that in DLY piglets (*p* < 0.05) (Figure 2B). Furthermore, immunohistochemical analysis of CD3, CD68, and IgA in the colon indicated that the relative protein expression levels of CD3, CD68, and IgA in the colonic mucosa of NX piglets were significantly higher than in DLY piglets (*p* < 0.05) (Figure 2C).

### 3.2. Differences in Colonic Metabolome Between NX Piglets and DLY Piglets

**Figure 3:**
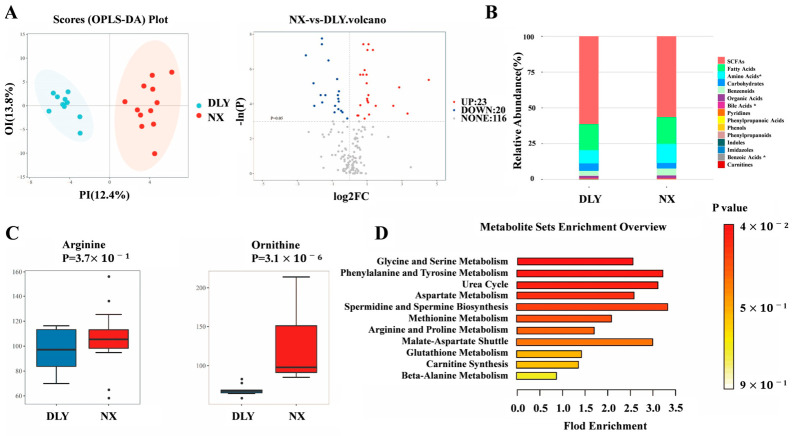
Differences in colonic metabolites between NX piglets and DLY piglets. (**A**) Analysis of metabolite OPLS-DA of colonic contents and Volcano plots of significantly changed metabolites (SCMs) among the two comparison groups; (**B**) stacked histogram of the top 30 metabolites’ abundance in colonic contents; (**C**) box diagram of the differential metabolites arginine and ornithine in colonic contents; (**D**) the SMPDB pathway analysis among NX and DLY piglets based on breed-associated differential metabolites. DLY, Duroc × Landrace × Yorkshire; NX, Ningxiang. * *p* < 0.05.

The OPLS-DA model revealed clear separation between breeds (Figure 3A), with 43 metabolites significantly altered (VIP > 1.0, *p* < 0.05; 23 upregulated, 20 downregulated in NX). As shown in Figure 3B,C, amino acid levels were significantly higher in NX piglets (*p* < 0.05), most notably ornithine (*p* < 0.01). Pathway enrichment analysis further demonstrated that these differential metabolites were associated with spermidine/spermine synthesis and arginine/proline metabolism pathways in NX piglets (Figure 3D), with key contributors including ornithine and putrescine.

### 3.3. Differences in Colonic Polyamine Metabolism Between NX and DLY Piglets

**Figure 4:**
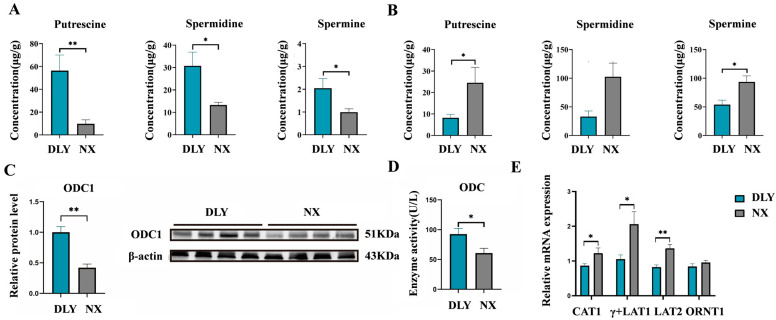
Differences in polyamine metabolism in the colon between NX piglets and DLY piglets. (**A**) Concentration of polyamines (putrescine, spermidine, and spermine) in colonic contents; (**B**) polyamine concentration in the colon; (**C**) ODC 1 protein expression in the colon; (**D**) ODC enzyme activity in the colon; (**E**) amino acid transport carrier mRNA expression level in the colon. ODC 1: ornithine decarboxylase 1; ODC: ornithine decarboxylase. DLY: Duroc × Landrace × Yorkshire; NX: Ningxiang. * *p* < 0.05, ** *p* < 0.01.

The concentrations of putrescine, spermidine, and spermine in colonic content were significantly lower in NX piglets than in DLY piglets (*p* < 0.05) (Figure 4A). Conversely, these polyamine concentrations in the colon were significantly higher in NX piglets compared to DLY piglets (*p* < 0.05) (Figure 4B). The relative protein expression level of ODC1, a rate-limiting enzyme for polyamine synthesis, was significantly higher in colonic tissues of DLY piglets than in NX piglets (*p* < 0.01) (Figure 4C). Additionally, colonic ODC enzyme activity was approximately 1.8-fold higher in DLY piglets than in NX piglets (*p* < 0.05) (Figure 4D). The mRNA expression levels of L-type amino acid transporter 2 (*LAT2*), γ^+^ L-type amino acid transporter 1 (*γ^+^LAT1*), and cationic amino acid transporter 1 (*CAT1*) in colonic tissue of NX piglets were also significantly higher than those in DLY piglets (*p* < 0.05) (Figure 4E).

### 3.4. Correlation Between Colonic Polyamine Levels and Intestinal Barrier

**Figure 5:**
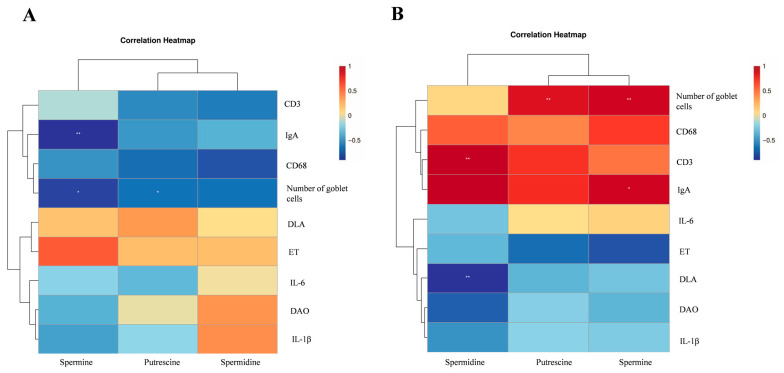
Correlation analysis of colonic polyamine levels and mucosal barrier between NX piglets and DLY piglets. (**A**) Analysis of the correlation between polyamines of colonic contents and mucosal barrier; (**B**) correlation analysis between colon polyamines and mucosal barrier. * *p* < 0.05, ** *p* < 0.01.

Next, the results showed that spermine in colonic content was significantly negatively correlated with colonic IgA expression, and putrescine and spermine levels in colonic content were significantly negatively correlated with the abundance of colonic goblet cells (*p* < 0.05) (Figure 5A). Spermidine levels in colonic tissue were found to be significantly positively correlated with colonic CD3 expression and significantly negatively correlated with plasma DLA concentration. The levels of putrescine and spermine in colonic tissue were significantly positively correlated with colonic goblet cells, and the levels of spermine were significantly positively correlated with IgA expression (*p* < 0.05) (Figure 5B).

### 3.5. ETEC-Induced Divergence in Colonic Polyamine Metabolism: NX-E vs. DLY-E Piglets

**Figure 6:**
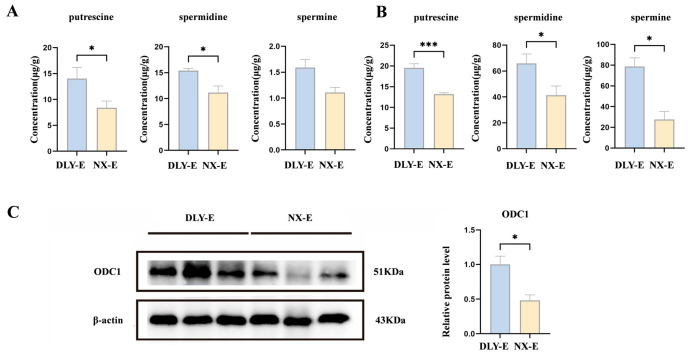
ETEC-induced divergence in colonic polyamine metabolism: NX-E vs. DLY-E piglets. (**A**) Concentration of polyamines (putrescine, spermidine, and spermine) in colonic content after ETEC infection; (**B**) polyamine concentration in the colon after ETEC infection; (**C**) ODC 1 protein expression in the colon after ETEC infection. DLY-E: Duroc × Landrace × Yorkshire-ETEC; NX-E: Ningxiang-ETEC. * *p* < 0.05, *** *p* < 0.001.

The colonic polyamine metabolism in NX-E and DLY-E piglets after ETEC challenge is shown in Figure 6. The concentrations of putrescine and spermidine in the colonic content of DLY-E piglets were significantly higher than those in NX-E piglets (*p* < 0.05) (Figure 6A). The concentrations of putrescine and spermine in the colon of NX-E piglets were higher than those of DLY-E piglets before ETEC challenge. However, after the ETEC challenge, the concentrations of putrescine in the colon of DLY-E piglets were significantly higher than those of NX-E piglets (*p* < 0.001) (Figure 6B), with spermidine and spermine concentrations also being significantly elevated in DLY-E piglets compared to NX-E piglets (*p* < 0.05) (Figure 6B). Furthermore, ODC1 protein expression in the colonic tissue of DLY-E piglets was significantly higher than in NX-E piglets (*p* < 0.05) (Figure 6C).

### 3.6. ETEC-Induced Colonic Barrier and Permeability Changes in NX-E vs. DLY-E Piglets

**Figure 7:**
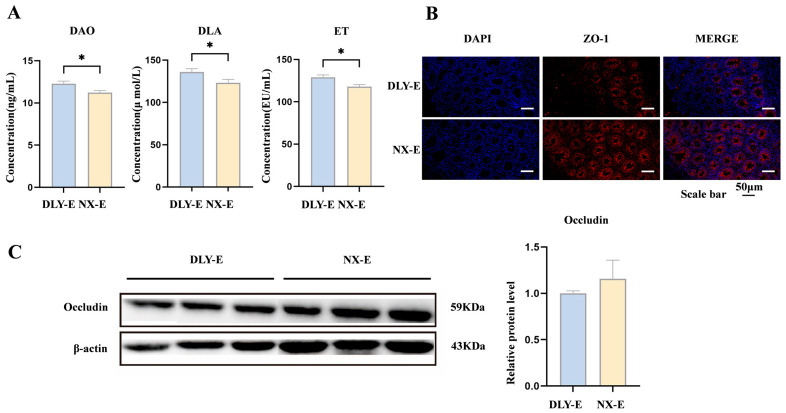
ETEC-induced colonic barrier and permeability changes in NX-E vs. DLY-E piglets. (**A**) DAO, DLA, and ET concentrations in plasma after ETEC infection. DAO, diamine oxidase; DLA, D-lactic acid; ET, endotoxin; (**B**) representative immunofluorescence images of tight junction protein (red: ZO-1) in the colon after ETEC infection (Scale bar, 50 μm); (**C**) occludin expression in the colon after ETEC infection. DLY-E, Duroc × Landrace × Yorkshire-ETEC; NX-E, Ningxiang-ETEC. * *p* < 0.05.

Plasma concentrations of DAO, DLA, and ET in NX-E piglets were significantly lower than those in DLY-E piglets after ETEC challenge (*p* < 0.05) (Figure 7A). The results of colonic immunofluorescence staining, shown in Figure 7B, demonstrate that the expression of ZO-1 protein in the colon of NX-E piglets was significantly higher than that in DLY-E piglets. Additionally, the relative protein expression level of occludin in the colon of NX-E piglets was numerically higher than that in DLY-E piglets, although the difference did not reach statistical significance (*p* > 0.05) (Figure 7C).

### 3.7. ETEC-Induced Divergent Colonic Immune-Inflammatory Responses in NX-E vs. DLY-E Piglets

**Figure 8:**
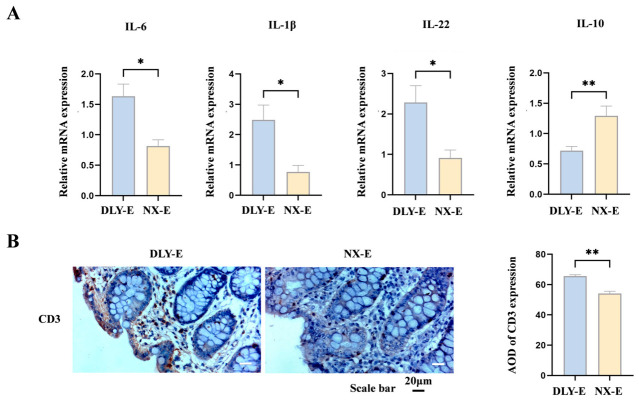
ETEC-induced divergent colonic immune-inflammatory responses in NX-E vs. DLY-E piglets. (**A**) The expression of *IL-1β*, *IL-22*, *IL-6*, and *IL-10* genes in the colon after ETEC infection; (**B**) representative immunohistochemical images and quantitative analysis of expression levels of immune cell proteins (CD 3) in the colon after ETEC infection (scale bar, 20 μm). DLY-E, Duroc × Landrace × Yorkshire-ETEC; NX-E, Ningxiang-ETEC. * *p* < 0.05, ** *p* < 0.01.

The relative mRNA expression levels of pro-inflammatory cytokines *IL-1β*, *IL-22*, and *IL-6* in the colon of NX-E piglets were significantly lower than in DLY-E piglets after ETEC challenge (*p* < 0.05); moreover, the relative mRNA expression level of the anti-inflammatory cytokine *IL-10* in the colon of NX-E piglets was markedly higher than that in DLY-E piglets (*p* < 0.01) (Figure 8A). These findings indicate a more pronounced inflammatory response in the colon of DLY-E piglets compared to NX-E piglets.

The immunohistochemical analysis of CD3 in the colon after ETEC challenge was shown in Figure 8B, where the relative protein expression level of CD3 in the colon of NX-E piglets was extremely significantly higher than that in DLY-E piglets (*p* < 0.01).

### 3.8. Distinct Colonic Microbiota Composition and Functional Potential in NX and DLY Piglets

**Figure A1:**
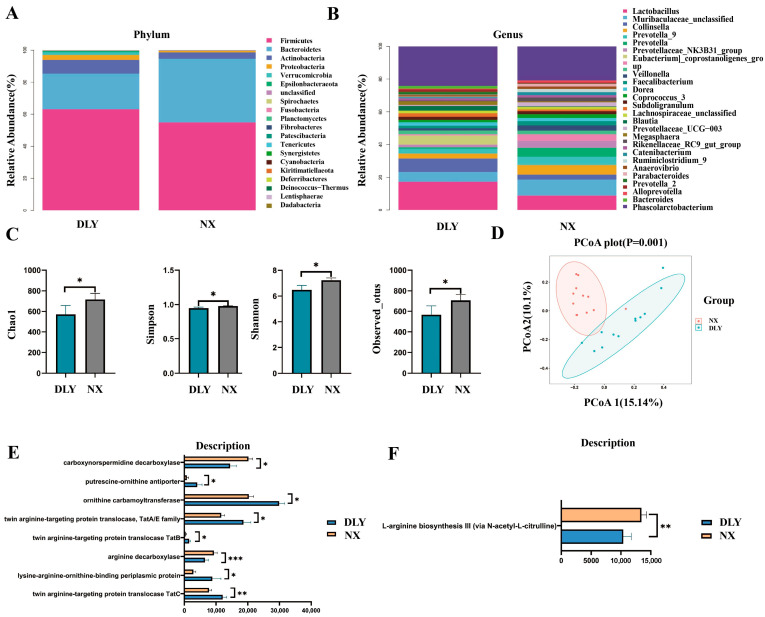
Differences in the colon microbial barrier between NX piglets and DLY piglets. (**A**) Columnar stacking graphs of the top 30 species in the horizontal abundance of colonic contents; (**B**) columnar stacks of the top 30 species in the horizontal abundance of colonic contents microorganisms; (**C**) results of microbial Alpha diversity in colonic contents; (**D**) PCoA analysis of colonic contents’ microorganisms; (**E**) difference analysis of colonic contents microbial function prediction of TIGRFAM; (**F**) analysis of PATHWAY differences in prediction of microbial function of colonic contents. DLY, Duroc × Landrace × Yorkshire; NX, Ningxiang. * *p* < 0.05, ** *p* < 0.01, and *** *p* < 0.001.

The richness and diversity of colonic microbial species in NX piglets were higher than those in DLY piglets (Figure A1C,D). At the phylum level (Figure A1A) [see Appendix A], the dominant bacterial phyla in both breeds were Firmicutes and Bacteroidetes. The relative abundance of Bacteroidetes was higher in the colon of NX piglets, whereas Actinobacteria and Proteobacteria were more prevalent in DLY piglets. At the genus level (Figure A1B), the relative abundances of *Muribaculaceae_unclassified*, *Prevotella_9*, *Prevotella*, *Prevotellaceae_NK3B31_group*, *Eubacterium_coprostanoligenes_group*, and *Faecalibacterium* were higher in the colon of NX piglets compared to DLY piglets, whereas *Lactobacillus*, *Collinsella*, and *Veillonella* were more abundant in the colon of DLY piglets. Predicted functional profiling based on 16S rRNA data revealed that pathways related to arginine decarboxylase and L-arginine biosynthesis III were significantly enriched in the colonic microbiota of NX piglets. In contrast, genes predicted to encode putrescine–ornithine antiporter, ornithine carbamoyl transferase, arginine transporter, and lysine–arginine–ornithine periplasmic binding protein were relatively more abundant in the colonic microbiota of DLY piglets (Figure A1E,F).

**Figure A2:**
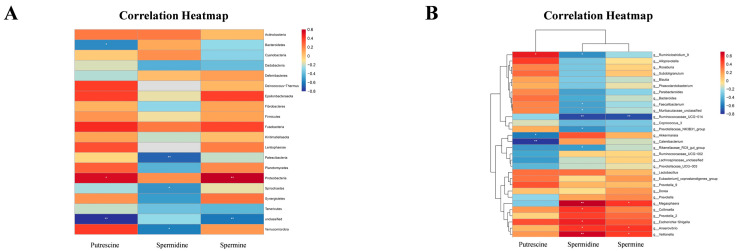
Correlation of colonic microbiota with polyamines: from phyla to genera. (**A**) Correlation analysis between the level of microbial phylum in colonic contents and polyamines; (**B**) correlation analysis between microbial genus level and polyamines in colonic contents. * *p* < 0.05, ** *p* < 0.01.

In colonic contents, putrescine exhibited a significant negative correlation with the relative abundance of Bacteroidetes, while spermidine correlated negatively with Verrucomicrobia at the phylum level. Conversely, both putrescine and spermine showed positive correlations with Proteobacteria (*p* < 0.05) (Figure A2A) [see Appendix A]. At the genus level, putrescine was negatively correlated with *Faecalibacterium* and unclassified *Muribaculaceae*, and spermidine demonstrated an inverse correlation with *Akkermansia* abundance. Significant positive correlations were observed between putrescine and the abundances of *Megasphaera*, *Anaerovibrio*, and *Escherichia-Shigella*, while spermine correlated positively with *Megasphaera* and *Anaerovibrio* (*p* < 0.05) (Figure A2B).

## 4. Discussion

Weaning stress in pig production often leads to intestinal inflammation and even diarrhea in piglets [34]. Consistent with this, Trial 2 showed a high incidence of diarrhea, with rates of 75% in DLY-E piglets and 25% in NX-E piglets. Goblet cells are part of the intestinal mucus layer and secrete abundant mucus, especially MUC2, which forms a protective barrier in intestinal mucus and prevents pathogenic microorganisms from adhering to intestinal epithelial cells [35]. In NX piglets, the number of colonic goblet cells increased, and the level of MUC2 expression increased, indicating a more intact colonic mucosal barrier [36]. Functionally, NX piglets exhibited lower plasma concentrations of intestinal permeability markers—diamine oxidase (DAO), D-lactate (DLA), and endotoxin (ET)—compared to DLY piglets, suggesting reduced intestinal permeability and more intact colonic barrier function. In addition to chemical and functional evidence, we evaluated the physical barrier by assessing tight junction proteins in the ETEC challenge trial (Trial 2). Following ETEC infection, NX-E piglets maintained significantly higher expression of ZO-1 and numerically higher occludin expression compared to DLY-E piglets, indicating better-preserved physical barrier integrity. These findings are consistent with previous studies. Consistent with these findings, Yi et al. (2021) demonstrated that ETEC K88 challenge downregulated both mRNA and protein expression of tight junction proteins (ZO-1, occludin, and claudin-1) in the jejunum and ileum of weaned piglets [37]. We also identified significant differences in the intestinal immune system and function between NX and DLY piglets. The findings from this study indicated that lower *IL-6* and *IL-1β* levels but higher *IL-10* levels were observed in NX piglets. *IL-6* and *IL-1β* are key pro-inflammatory cytokines whose levels are closely associated with the intensity of the intestinal inflammatory response. Typically, when injury or infection occurs within the intestine, there is a marked increase in the expression of *IL-6* and *IL-1β*, which further promotes and exacerbates inflammatory responses [38,39,40]. *IL-10* primarily functions as an anti-inflammatory cytokine by inhibiting the production of pro-inflammatory mediators such as *IL-1β* [41]. The high expression levels of *IL-10* within the colon serve to suppress excessive inflammatory responses while protecting against potential damage to intestinal tissues [42,43,44]. Intestinal *IL-1β*/*IL-6*–*IL-10* balance governs immune homeostasis via T cell (Th17/Treg), macrophage (M1/M2), and B cell (Breg) regulation [45,46,47]. Our further investigation revealed that the relative protein expression levels of CD3 (a T cell marker), CD68 (a macrophage marker), and IgA (a B cell marker) in the colons of NX piglets were significantly higher than those observed in DLY piglets. This finding underscores the maturity and efficacy of the intestinal mucosal immune barrier function in NX piglets. The synergistic action of these immune cells within the intestinal mucosal barrier not only enhances the resistance of NX piglets to intestinal pathogens but also supports a more effective immune response to intestinal challenges.

The homeostasis of polyamines in the intestinal environment is complicated and mainly regulated by the synthesis, transport, metabolism, and storage of polyamines and their precursors [48]. Previous studies have suggested that gut microorganisms are potential sources of colonic polyamines, but the levels of polyamines in colonic contents may not directly reflect those in colonic tissue [49]. The results of this study provide direct evidence for this complex relationship: NX piglets had higher concentrations of polyamines in the colon tissue than DLY piglets, whereas they had lower concentrations of polyamines in the colon contents than DLY piglets. This negative correlation may reflect the complex regulatory mechanism of polyamine metabolism, including the storage capacity of polyamines, metabolic differences, and transport efficiency. At the same time, we found that the relative protein expression of ODC1 and ODC activity in the colon tissues of DLY piglets was higher compared to those of NX piglets. One possible interpretation is that microbial polyamine metabolism in the colon is more active in DLY piglets following weaning stress [50], potentially facilitating intestinal repair. However, this interpretation remains speculative without direct functional measurements. The observed differences in colonic polyamine metabolism between NX and DLY piglets were associated with distinct microbial community structures. Specifically, NX piglets harbored higher microbiota richness and diversity, with higher relative abundances of Bacteroidetes and several *Prevotella*-related taxa. Predicted functional profiling suggested that the colonic microbiota of NX piglets was enriched for the arginine decarboxylase and L-arginine biosynthesis III pathway, which could promote arginine accumulation. In contrast, the microbiota of DLY piglets showed higher predicted abundances of genes encoding putrescine–ornithine antiporter, ornithine carbamoyl transferase, arginine transporter, and lysine–arginine–ornithine periplasmic binding protein. Ornithine carbamoyl transferase is a key enzyme that links ornithine and citrulline, providing the necessary precursor substances for the synthesis of arginine [51]. And the L-arginine biosynthesis III pathway synthesizes arginine through the intermediate of N-acetyl-L-citrulline [15]. Arginine decarboxylase catalyzes the decarboxylation of arginineand participates in putrescine synthesis [52]. The putrescine–ornithine antiporter and arginine translocase play pivotal roles in the transport of polyamines and their precursors, providing the necessary spatial or temporal coordination for polyamine synthesis, and thus promoting the synthesis and metabolism of polyamines [53,54]. It is particularly worth noting that when the putrescine—ornithine reverse transporter works, microorganisms excrete putrescine in exchange for ornithine, which explains why the colon contents of DLY piglets accumulate putrescine but the colon tissues have insufficient polyamines. The metabolism of polyamines in the gut is crucial for maintaining the integrity of the intestinal mucosa and immune responses [55,56,57]. The accumulation of arginine, as the substrate for the starting point of polyamine synthesis, is the rate-limiting step in polyamine synthesis [58]. An increase in ornithine further accelerates the formation of putrescine and converts spermidine to spermine through spermine synthase [59,60,61]. Based on differences in polyamine metabolism, NX piglets exhibited higher levels of arginine and ornithine than DLY piglets, with more enriched pathways for spermidine and spermine synthesis and arginine and proline metabolism in NX piglets. This suggests that NX piglets maintain colon tissue polyamine homeostasis through enhanced absorption and utilization of arginine and ornithine in colonic tissues. The content of polyamines in the tissues of DLY piglets is relatively low, and they rely on colonic microorganisms to synthesize and supplement polyamines. In addition, we also found that compared with NX piglets, the relative protein expression level of ODC1 and the activity of ODC in the colon tissue of DLY piglets were both higher. Combining the differences in the two piglet polyamine metabolic pathways discussed earlier, after weaning stress, the polyamine metabolism of colonic microorganisms in DLY piglets is more active [50]. One possible interpretation is that this active state may promote the repair of intestinal damage, but alternative explanations are equally plausible.

Especially in our ETEC challenge model, weaning stress had more significant effects on polyamine metabolism and intestinal barrier function. The polyamine concentration and ODC1 protein expression in the colons of DLY piglets were higher than those of NX piglets, which suggested the colon polyamine metabolism of DLY piglets was more active after ETEC infection, while that of NX piglets remained relatively stable. This may reflect greater stress resistance in NX piglets, although direct measurements of stress responses (e.g., cortisol levels) are needed to confirm this. Weaning stress-induced surges in plasma cortisol levels increase ODC activity by 230%, and polyamine synthesis by 72~157% in the jejunum [62], while relatively lower cortisol levels were found in piglets during weaning [8]. Compared to DLY piglets, NX piglets showed reduced ODC1 protein expression, ODC activity, and lower polyamine levels in colonic contents. Taken together, these observations suggest that polyamine metabolism in the colon and colonic microbiota may be more active in DLY piglets, although this interpretation requires functional validation.

In colonic contents, both putrescine and spermine showed positive correlations with Proteobacteria. At the genus level, positive correlations were observed between putrescine and *Escherichia-Shigella* abundances. Notably, Proteobacteria and *Escherichia-Shigella* contribute to polyamine synthesis [15]. The polyamines’ metabolic activities effectively affect the activation, proliferation, and differentiation of immune cells [63]. Polyamines could effectively inhibit the secretion and release of *TNF-α*, *IL-1*, *IL-6*, *CCL3*, and *CCL4* in LPS-induced macrophages [64]. In this study, polyamine levels in the colon were negatively correlated with the pro-inflammatory factors, and combined with high polyamines and low pro-inflammatory factors in the colons of NX piglets. However, this association manifested differently in the colon and in the colon content, where polyamine levels in colon content were inversely associated with colonic goblet cells and IgA. Conversely, spermidine levels in colonic tissue exhibited a negative correlation with plasma DLA. Polyamines may inhibit intestinal leakage of DLA, DAO, and ET by enhancing intestinal barrier function [65]. While previous studies have shown that ODC overexpression inhibits pro-inflammatory cytokine secretion [61,62,63,64], whether the elevated ODC expression observed in DLY piglets represents a similar protective mechanism remains unclear.

We further investigated differences in intestinal immune responses between the two breeds following ETEC infection. After ETEC infection, plasma concentrations of these markers were lower, but the tight junction protein expressions were higher in NX piglets compared to DLY piglets, indicating less severe intestinal barrier damage. This resilience may be related to the higher intestinal immune maturity of NX piglets. The observed differences in inflammatory factors and CD3 expression are consistent with this interpretation, but further studies are needed to establish causality. NX piglets exhibited higher protein expression levels of CD3 and mRNA expression levels of *IL-10* in their colons, alongside lower mRNA expression levels for *IL-1β*, *IL-22*, and *IL-6*. These results indicated that the intestinal mucosal immunity in NX piglets was more comprehensive and could respond more effectively to ETEC challenges while safeguarding intestinal tissue from potential damage.

Several limitations should be acknowledged. First, the NX and DLY piglets originated from two different commercial farms prior to the experiment; therefore, farm-related factors (e.g., diet, microbiota exposure, or management practices) may confound the observed breed differences. Second, the functional predictions based on 16S rRNA data are computational inferences, not direct measurements of microbial metabolic activity, and correlation analyses do not imply causation; thus, our microbiota findings are hypothesis-generating rather than definitive. Third, in the ETEC challenge experiment (Trial 2), polyamine levels and ODC expression were measured only at a single time point (72 h post-challenge); dynamic measurements (e.g., time-course sampling or metabolic flux analysis) would provide a more comprehensive understanding of polyamine metabolic stability. Fourth, this study compared only two breeds, used a single reference gene (*β-actin*) for RT-qPCR without formal validation, and did not establish a direct concentration-dependent relationship between polyamine levels and immune function. Future studies should include additional breeds, employ multiple reference genes (e.g., GAPDH, TBP), conduct dose–response experiments, and incorporate dynamic measurements to address these limitations.

## 5. Conclusions

In summary, compared with DLY piglets, NX piglets have more mature colon immune function and stronger stability in the face of ETEC challenge. The higher polyamine levels in colonic tissue of NX piglets under baseline conditions, together with their more stable polyamine levels following ETEC infection, suggest that NX piglets may possess a well-developed intestinal mucosal system that has the potential to be more resilient to stress. In contrast, DLY piglets showed a more significant increase in the activity of polyamine metabolism when exposed to external stimuli, such as ETEC challenge, which may reflect a more vulnerable gut barrier and an attempted compensatory repair response. However, this study has not yet established the specific polyamine concentration range that supports intestinal immune stability in piglets, and the universality of this observation across different pig breeds requires further validation.

## Figures and Tables

**Table 1:** Basic diet composition and nutrient level of weaned piglets (%, air-dried basis).

Items, %	Contents
Corn	57.60
Soybean meal	5.70
Peanut meal	9.20
Wheat bran	2.10
Rice bran	13.70
Soybean oil	5.70
CaHPO4	0.53
Limestone	0.88
L-Lysine	0.18
L-Threonine	0.21
Rice Mill Feed	3.20
Premix ^1^	1.00
Total	100
Nutrient composition ^2^	
DE, MJ/kg	14.33
CP	17.75
EE	3.73
ADF	7.39
NDF	18.11
Ash	4.95
Ca	0.55
P	0.47

^1^ Premix per kg diet: Cu, 0.05 mg; I, 0.03 mg; Fe, 0.8 mg; Se, 0.002 mg; Zn, 0.8 mg; Mn, 0.06 mg; vitamin K (menadione), 1.3 mg; vitamin B_1_, 2 mg; vitamin B_2_, 5.8 mg; vitamin B_3_, 18.79 mg; vitamin B_12_, 14.5 μg; vitamin A, 3324 IU; vitamin D, 376 IU; vitamin E, 28.9 IU; choline chloride, 80 mg; antioxidants, 200 mg; andfungicide, 500 mg. ^2^ Calculation of digestible energy, crude protein, calcium, and total phosphorus content. CaHPO4: Calcium hydrogen phosphate; DE: Digestible energy; MJ/kg: Megajoules per kilogram; CP: Crude protein; EE: Ether extract; ADF: Acid detergent fiber; NDF: Neutral detergent fiber; Ca: Calcium; P: Phosphorus. All nutrient composition values were determined by proximate analysis (AOAC, 2019) and are presented as analyzed values on an as-fed basis [26].

**Table 2:** Primer sequences used for quantitative reverse transcription-PCR.

Genes	Primer	Sequences (5′–3′)	Product Size.bp	Accession Number
*β-actin*	Forward	CTGCGGCATCCACGAAACT	147	XM_021086047.1
	Reverse	AGGGCCGTGATCTCCTTCTG		
*IL-6*	Forward	AAATGTCGAGGCCGTGCAGATTAG	86	JF906513.1
	Reverse	GGGTGGTGGCTTTGTCTGGATTC		
*IL-1β*	Forward	CAGCCATGGCCATAGTACCT	216	XM_021085847.1
	Reverse	CCACGATGACAGACACCATC		
*IL-10*	Forward	GGGCTATTTGTCCTGACTGC	105	HQ026020.1
	Reverse	GGGCTCCCTAGTTTCTCTTCC		
*IL-22*	Forward	GATGAGAGAGCGCTGCTACCTGG	112	XM_021091968.1
	Reverse	GAAGGACGCCACCTCCTGCATGT		
*LAT2*	Forward	TATGAGCAGAGGAGCCTGGAGAATC	134	XM_005661967.3
	Reverse	AGCAGCTTGTCCTTCCTTGTTGATG		
*CAT1*	Forward	TGCCCATACTTCCCGTCC	192	XM_021065165.1
	Reverse	GGTCCAGGTTACCGTCAG		
*ORNT1*	Forward	ACTGCTGCCTCAAGACCTACTCG	95	XM_021065130.1
	Reverse	GACGGAGTTCTCGGCGATGTTG		

*IL-6*, Interleukin-6; *IL-1β*, Interleukin-1 beta; *IL-10*, Interleukin-10; *IL-22*, Interleukin-22; *LAT2*, L-type amino acid transporter 2; *CAT1*, cationic amino acid transporter 1; *ORNT1,* Ornithine Transporter 1.

## Data Availability

The original contributions presented in this study are included in the article. Further inquiries can be directed to the corresponding author.

## Abbreviations

NX	Ningxiang
DLY	Duroc × Landrace × Yorkshire
ETEC	*Enterotoxigenic Escherichia coli*
DAO	Diamine oxidase
DLA	D-lactate
ET	Endotoxin
ODC	Ornithine decarboxylase
ELISA	Enzyme-linked immunosorbent assay
RT-qPCR	Real-time quantitative polymerase chain reaction
H&E	Hematoxylin and eosin
IHC	Immunohistochemistry
IF	Immunofluorescence
*IL-6*	Interleukin-6
*IL-1β*	Interleukin-1 beta
*IL-10*	Interleukin-10
*γ^+^LAT1*	γ^+^ L-type amino acid transporter 1
*CAT1*	Cationic amino acid transporter 1
